# Two siblings with lipoid congenital adrenal hyperplasia: first reported case in Serbia and literature review

**DOI:** 10.3389/fendo.2026.1912249

**Published:** 2026-08-19

**Authors:** Maja D. Jesic, Smiljka Kovacevic, Vera Zdravkovic, Sonja Pavlovic, Anita Skakic, Vladimir Gasic, Marija Mijovic, Milos M. Jesic, Ljiljana Lukic

**Affiliations:** 1 Endocrinology Department, University Children’s Hospital, Belgrade, Serbia; 2 Faculty of Medicine, University of Belgrade, Belgrade, Serbia; 3 Institute of Molecular Genetics and Genetic Engineering, University of Belgrade, Belgrade, Serbia; 4 Genetic Department, University Children’s Hospital, Belgrade, Serbia; 5 Neonatology Department, University Children’s Hospital, Belgrade, Serbia; 6 Clinic for Endocrinology, Diabetes and Metabolic Diseases, University Clinical Center of Serbia, Belgrade, Serbia

**Keywords:** children, lipoid congenital adrenal hyperplasia, primary adrenal insufficiency, steroidogenic acute regulatory gene, steroidogenic acute regulatory protein

## Abstract

**Background:**

Lipoid congenital adrenal hyperplasia (LCAH) is an uncommon but life-threatening autosomal recessive disorder. It is caused by a mutation in the steroidogenic acute regulatory protein (StAR), encoded by the *STAR* gene.

**Methods:**

Clinical manifestations of two siblings with primary adrenal insufficiency (PAI) were described. A female infant was admitted to the hospital at six months of age for vomiting and failure to thrive. The diagnosis of PAI of unknown etiology was established. The girl’s younger brother was diagnosed with PAI at the age of 1.5 years during a febrile illness. His external genitalia showed undescended testes, scrotal hypoplasia, and hypospadia. Their family history was normal, and both parents were healthy. Clinical exome sequencing was used to identify mutations in candidate genes.

**Results:**

Two heterozygous *STAR* mutations, p.Trp250Ter and p.Arg188Cys, were identified in both children. The mother was heterozygous for the p.Trp250Ter mutation, and the father was heterozygous for the p.Arg188Cys mutation.

**Conclusion:**

Glucocorticoid, mineralocorticoid, and sex hormone therapy should be individualized according to the clinical presentation and laboratory findings. Because genotype and phenotype do not correlate consistently in LCAH, therapy and follow-up must be individualized.

## 1 Introduction

Lipoid congenital adrenal hyperplasia (LCAH, OMIM #201710, OMIM - Online Mendelian Inheritance in Man) is an uncommon but life-threatening autosomal recessive disorder. It is caused by a mutation in the steroidogenic acute regulatory protein (StAR), encoded by the *STAR* gene located on chromosome 8p11.2 (1). To date, more than 70 *STAR* variants have been reported (2), mostly in Caucasian children. The most frequently reported *STAR* variants differ among populations: c.772C>T in Chinese populations (2), c.545G>A in eastern Saudi Arabia (3), c.545G>T among Palestinian Arabs (3, 4). The c.772C>T variant in the *STAR* gene is the most commonly reported variant in Japanese and Korean populations, with an estimated incidence of approximately 1 in 250,000 to 1 in 300,000 newborns (3). In European populations, the relatively rare *STAR* variants, c.749G>A and c.562C>T, have been reported most frequently.

StAR is a cytoplasmic enzyme that transfers cholesterol to the inner mitochondrial membrane, and it is involved in the first steps of steroidogenesis in the adrenal cortex, gonads, kidneys, and brain. Clinical presentation can vary, depending on enzyme activity, from classic LCAH, defined by early-onset deficiency of glucocorticoids, mineralocorticoids, and sex steroids, to non-classic LCAH, characterized by late-onset glucocorticoid deficiency (5, 6). The study aimed to present the clinical course, diagnosis, and treatment of siblings with LCAH, the first cases diagnosed in Serbia, and to review the relevant literature.

## 2 Methods

### 2.1 Patients

Both patients, a sister and a brother, underwent clinical examination with assessment of growth and sexual development. Clinical data included birth measurements and birth history; family history was obtained. Routine laboratory analyses included adrenocorticotropic hormone (ACTH), cortisol, 17-hydroxyprogesterone, plasma renin activity (PRA), androstenedione, and testosterone. Genetic testing was performed, and written informed consent was obtained from both parents.

### 2.2 Molecular assays

Genomic DNA was extracted from peripheral blood using the DNA extraction kit (Qiagen, Hilden, Germany). Clinical exome sequencing was performed using the TruSight One panel (Illumina), which targets the coding exons and exon–intron boundaries of 4,813 clinically relevant genes selected based on their established associations with human genetic disorders, as curated from OMIM, ClinVar, HGMD, and current clinical literature. Sequencing reads were aligned to the human reference genome (hg19). Variant analysis was initially focused on the congenital adrenal hyperplasia (CAH) gene panel (HP:0008258, Human Phenotype Ontology), with particular emphasis on CYP21A2, the most common genetic cause of CAH. Because of the high sequence homology between CYP21A2 and its pseudogene CYP21A1P, gene conversions and large deletions were additionally assessed by gene-specific PCR amplification, as previously described (7–9). Since no causative variants were identified in CYP21A2, the remaining genes in the CAH panel, along with all other genes covered by the TruSight One panel, were systematically evaluated. Common variants (minor allele frequency >1%) reported in the gnomAD Genomes, gnomAD Exomes, and 1000 Genomes databases were excluded. All pathogenic, likely pathogenic, and variants of uncertain significance (VUS) relevant to the patient’s phenotype were systematically reviewed. Variant interpretation and prioritization were performed using VarSome Professional (Saphetor SA, Lausanne, Switzerland) (10), taking into account the inheritance pattern, gene–disease association, available literature, and in silico predictions within the ACMG/AMP variant interpretation framework. Familial segregation of the identified STAR variants was evaluated using clinical exome sequencing data from both parents.

### 2.3 Literature review

LCAH was searched in the PubMed database for European articles. All variants in the *STAR* gene from publications were classified and interpreted in accordance with the ACMG (American College of Medical Genetics and Genomics)/AMP (Association for Molecular Pathology) standards and guidelines, using a five-tier system (P, LP, VUS, LB, B) based on evidence from population, computational, functional, and segregation data (11).

### 2.4 Statistical analysis

All data were analyzed using Microsoft Excel software.

## 3 Results

### 3.1 Case presentations

#### 3.1.1 Patient 1

A Serbian female infant was born at 40 gestational weeks with a normal birth weight of 3710 g, a birth length of 54 cm, and normal external female genitalia without signs of virilization. She was treated with phototherapy for neonatal unconjugated hyperbilirubinemia. At six months of age, she was admitted to the hospital for vomiting and failure to thrive. Her length was 70 cm (95^th^ percentile), weight was 6650 g (25^th^ percentile), and external genitalia were of a normal female. Laboratory analyses showed hyponatremia at 123 mmol/l, hyperkalemia at 7 mmol/l, and normoglycemia at 5.3 mmol/l (**Table 1**). Basal cortisol was 8.8 µg/dl (normal 2.97-23.4 µg/dl). The ACTH stimulation test showed no significant rise in cortisol levels (0 minute 8.8 ug/dl, 30 minute 8.9 ug/dl, 60 minute 9 ug/dl), while 17-hydroxyprogesterone levels were normal (0 minute 0.42 ng/ml, 30 minute 0.43 ng/ml, 60 minute 0.33 ng/ml). Karyotype was female 46, XX. The diagnosis of primary adrenal insufficiency (PAI) of unknown etiology was established. Hydrocortisone and fludrocortisone substitutional therapy was started.

**Table 1:** Laboratory results of patient 1.

Analyses	Result	Normal range
Natrium (mmol/l)	123	135-145
Potassium (mmol/l)	7	3.5-5
Glycemia (mmol/l)	5.3	3.6-6.1
Cortisol (µg/dl)	8.8	2.97-23.4
PRA (ng/ml/h)	11	0.3-3.9
Aldosterone (ng/dl)	24.5	3-35
17-hydroxyprogesterone (ng/ml)	0.42	<2
ACTH stimulation test (0 minute)		
Cortisol (µg/dl)	8.8	2.97-23.4
17-hydroxyprogesterone (ng/ml)	0.33	<2
ACTH stimulation test (60 minute)		
Cortisol (µg/dl)	9	>18
17-hydroxyprogesterone (ng/ml)	0.43	<9.8

PRA, plasma renin activity; ACTH, adrenocorticotropic hormone.

Clinical exome sequencing revealed two heterozygous pathogenic variants in the *STAR* gene, c.749G>A(p.Trp250Ter) and c.562C>T(p.Arg188Cys), that are associated with LCAH.

#### 3.1.2 Patient 2

The girl’s younger brother was born at 40 gestational weeks with a normal birth weight of 3920g and a birth length of 57cm, Apgar score 9. He was referred to a urologist for examination at the age of three months due to undescended testes, scrotal hypoplasia, and hypospadias. Ultrasound showed both testes were located in the inguinal canals, so he was followed up for the next period. At the age of 1.5 years, he was hospitalized due to an electrolyte imbalance (hyponatremia 125 mmol/l, hyperkalemia 6.1 mmol/l, hypoglycemia 2.7 mmol/l) during a febrile respiratory illness (**Table 2**). Laboratory analyses showed a low cortisol level of 5.2 µg/dl (normal 2.97-23.4 µg/dl), a normal 17-hydroxyprogesterone level of 0.6 ng/ml (normal range <2 ng/ml), a high PRA of 19 ng/ml/h (normal range 0.3-3.9), a low androstenedione of 0.11 ng/ml (normal range 0.15-3.1 ng/ml), and a low testosterone level of 0.06 ng/ml (normal range 1.75-7.81 ng/ml). Karyotype was normal male 46, XY, and adrenal ultrasound was described as normal. PAI was diagnosed, so hydrocortisone and fludrocortisone therapy was started. At the time of diagnosis, the boy was of normal height, 84 cm (75^th^ percentile), and weight 10 kg (7^th^ percentile). Clinical exome sequencing showed the same result as in the sister, compound heterozygosity for pathogenic variants in the *STAR* gene.

**Table 2:** Laboratory results of patient 2.

Analyses	Result	Normal range
Natrium (mmol/l)	125	135-145
Potassium (mmol/l)	6.1	3.5-5
Glycemia (mmol/l)	2.7	3.6-6.1
Cortisol (µg/dl)	5.2	2.97-23.4
PRA ng/ml/h	19	0.3-3.9
Aldosterone (ng/dl)	22.4	3-35
17-hydroxyprogesterone (ng/ml)	0.6	<2
Androstenedione (ng/ml)	0.11	0.15-3.1
Testosterone (ng/ml)	0.06	1.75-7.81

PRA, plasma renin activity; ACTH, adrenocorticotropic hormone.

**Figure 1:**
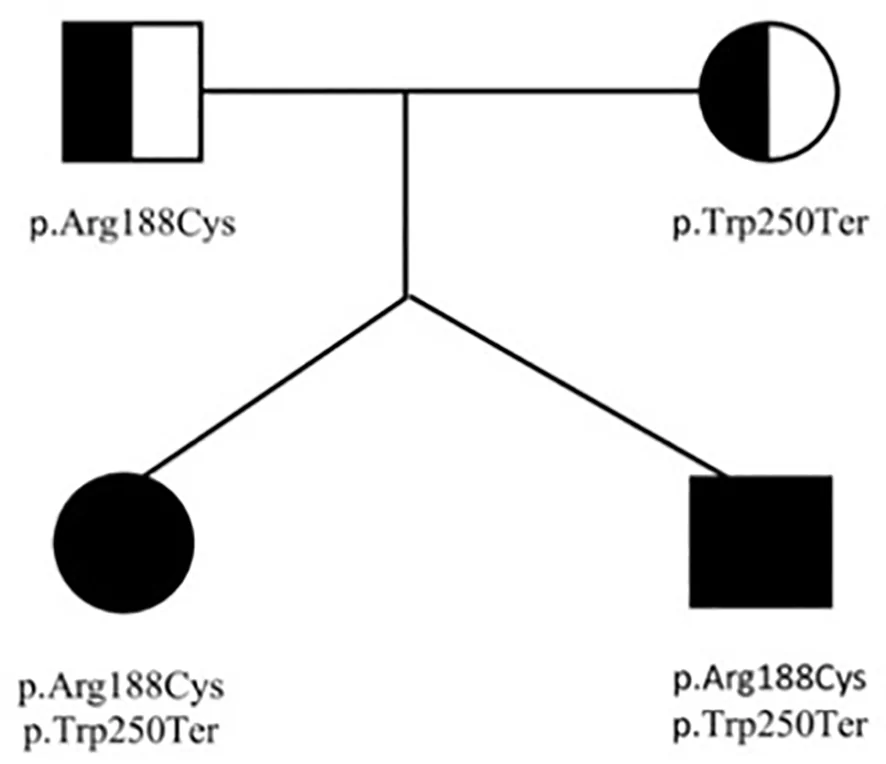
Pedigree of family with STAR mutations. Unaffected parents are heterozygous (half-filled symbols), and children are compound heterozygous for mutations (black symbols).

Neither parent showed any symptoms of LCAH. The mother was heterozygous for the p.Trp250Ter mutation, and the father was heterozygous for the p.Arg188Cys mutation (**Figure 1**).

**Figure 2:**
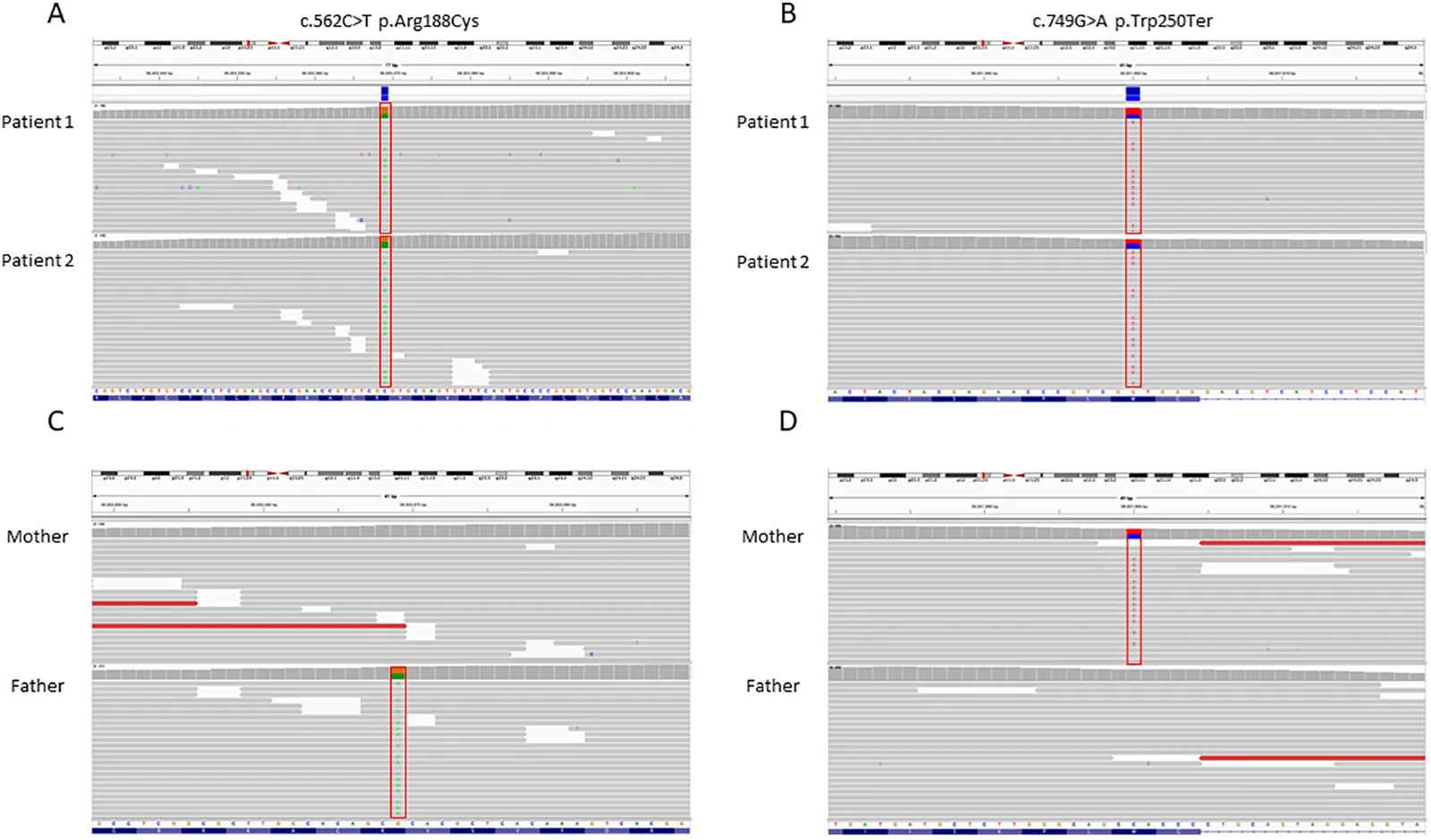
IGV screenshots illustrating the two pathogenic *STAR* variants identified by clinical exome sequencing. **(A)** The c.562C>T (p.Arg188Cys) variant in both affected siblings. **(B)** The c.749G>A (p.Trp250Ter) variant in both affected siblings. **(C)** Familial segregation of the c.562C>T (p.Arg188Cys) variant, demonstrating its presence in the affected siblings and their father. **(D)** Familial segregation of the c.749G>A (p.Trp250Ter) variant, demonstrating its presence in the affected siblings and their mother. The segregation pattern is consistent with compound heterozygosity in both affected siblings. All IGV screenshots are shown in the forward-strand orientation; therefore, the displayed reference and alternate nucleotides correspond to the forward genomic strand.

Representative IGV screenshots of both variants in the affected siblings, together with their familial segregation, are shown in **Figure 2**.

#### 3.1.3 Follow-up

The patients attended regular endocrinology follow-up visits every 6 months. Their growth was appropriate for age. They were treated with hydrocortisone (∼10 mg/m2/day) and fludrocortisone (0.05-0.1 mg/day), and their blood pressure, sodium, potassium, and PRA levels remained normal.

The boy underwent right orchiopexy at the age of four years, because he had migratory testicle. At 5.5 years of age, his height was 114 cm (50-75^th^ percentile), and his weight was 17 kg (10^th^ percentile). Physical examination showed that both testes were in the scrotum, with a volume of 2 mL each.

The girl was last seen at 9 years of age. Her height was 134 cm (50-75^th^ percentile), and her weight was 34.5 kg (75-90^th^ percentile). She had no clinical signs of puberty. During follow-up, patients did not develop adrenal crisis.

### 3.2 Literature review

All European-published articles on LCAH in PubMed were reviewed.

**Table 3** shows 19 patients with reported mutations in the *STAR* gene, diagnosed in the European population, including the present cases. Eight 46, XX and eleven 46, XY patients are presented. 10 different variants in the *STAR* gene were reported. Two 46, XY patients had hypospadia or cryptorchidism, one had undifferentiated external genitalia, and seven had normal female genitalia. The age at presentation was from the first day of life to 1,5 year old.

**Table 3:** Summary of pediatric patients diagnosed with LCAH in Europe.

Number	Gender	Age	Presentation	External genitalia	Kariotype	Variants of *STAR* gene (ACMG/AMP class)	Reference
1.	F	3 weeks	Vomiting, hyperpigmentation, inguinal hernia, PAI	Normal female	46, XY	Homozygosity: c.749G>A, p.Trp250Ter (P)	Korsch E et al.,1999 (12).
2.	F	5 months	PAI	Normal female	46, XY	Homozygosity: c.779T>C, p.Leu260Arg (P)	Fluck CE et al., 2005 (13).
3.	F	2,5 months	Failure to thrive	Normal female,	46, XY	Compound heterozygosity: c.779T>C, p.Leu260Arg (P) and c.470T>C, p.Leu157Pro (LP)	Fluck CE et al., 2005 (13).
4.	F	3,5 months	Vomiting during viral infection	Normal female	46, XY	Compound heterozygosity: c.779T>C, p.Leu260Arg (P) and c.629_630delCT, p.Leu210fsTer39 (P)	Fluck CE et al., 2005 (13).
5.	F	infant	PAI	DSD	46, XY	Homozygosity: c.444C>A, p.Asn148Lys (LP)	Bens Set al., 2010 (14).
6	M	3,5 months	Prolonged jaundice Pigmentation, ambiguous genitalia, PAI	Micropenis cryptorchidism	46, XY	Compound heterozygosity: c.562C>T, p.Arg188Cys (P) and c.749G>A, p.Trp250Ter (P)	Sahakitrungruang T et al., 2011 (15).
7.	F	1 day	PAI	Normal female	46, XX	Homozygosity: c.466-1G>A, p.Val156GlyfsTer19 (P)	Camats N et al., 2013 (16).
8.	F	8 months	Failure to thrive, PAI	Normal female	46, XX	Homozygosity: c.834del11bp, p.Lys236delfsTer43 (P)	Sertedaki A et al., 2013 (17).
9.	F	2 months	PAI	Normal female	46, XX	Homozygosity: c.834del11bp, p.Lys236delfsTer43 (P)	Sertedaki A et al., 2013 (17).
10.	F	20 days	PAI	Normal female	46, XX	Homozygosity: c.834del11bp, p.Lys236delfsTer43 (P)	Sertedaki A et al., 2013 (17).
11.	F	25 days	PAI	Normal female	46, XY	Homozygosity: c.749G>A, p.Trp250Ter (P)	Sertedaki A et al., 2013 (17).
12.	F	16 days	PAI	Normal female	46, XY	Homozygosity: c.749G>A, p.Trp250Ter (P)	Sertedaki A et al., 2013 (17).
13.	F	2.4 months	PAI	Normal female	46, XY	Compound heterozygosity: c.444C>A, p.Asn148Lys (LP) and c.577C>T, p.Arg193Ter (P)	Kaur J et al., 2016 (18).
14.	F	7 months	PAI	Normal female	46, XX	Compound heterozygosity: c.562C>T, p.Arg188Cys (P) and c.577C>T, p.Arg193Ter (P)	Bizzarri C et al., 2017 (19).
15.	F	14 months	hyperpigmentation	Normal female	46, XX	Homozygosity: c.562C>T, p.Arg188Cys (P)	Burget L et al., 2018 (20).
16.	M	Neonatal period	PAI	ND	46, XY	Homozygosity: c.562C>T, p.Arg188Cys (P)	Burget L et al., 2018 (20).
17.	F	1,5 month	PAI	Normal female	46, XX	Compound heterozygosity: c.611C>G, p.Thr204Arg (VUS) and c.779T>G, p.Leu260Arg (P)	Katharopoulos E et al, 2020 (21).
18.	F	6 months	Failure to thrive, PAI	Normal female	46, XX	Compound heterozygosity: c.749G>A, p.Trp250Ter (P) and c.562C>T, p.Arg188Cys (P)	Present case
19.	M	1,5 years	PAI due to infection	Cryptorchidism Hypospadia	46, XY	Compound heterozygosity: c.749G>A, p.Trp250Ter (P) and c.562C>T, p.Arg188Cys (P)	Present case

F, female; M, male. ACMG, American College of Medical Genetics and Genomics; AMP, Association for Molecular Pathology; PAI, primary adrenal insufficiency; DSD, disorder of sex development; ND, not described.

## 4 Discussion

### 4.1 Phenotype and genotype of the LCAH

StAR is important for steroid biosynthesis in the adrenal cortex and gonads. A mutation in the *STAR* gene causes a partial or complete loss of StAR protein function; therefore, the clinical presentation of LCAH depends on StAR activity. Clinically, it presents as PAI and 46, XY disorder of sex development, or as PAI and 46, XX with normal female external genitalia (1).

Complete loss of enzyme activity causes the most severe form of the disease, classic LCAH, which manifests in the neonatal period or early infancy. Mutations affecting the C-terminal functional domain of StAR severely impair StAR function (4, 22). The C-terminal domain is a cholesterol-binding site that is crucial for the protein’s biological activity and essential for steroidogenesis. As a result, StAR-dependent steroidogenesis is diminished.

Partial StAR activity, usually retaining 10-25% of enzyme activity, is responsible for a less severe form of disease, non-classic LCAH, which is characterized by later onset and an initial manifestation of isolated adrenal insufficiency. Lu et al. concluded that the age of PAI onset is not a key determinant for differentiating between classic and non-classical forms instead;one should rely on the percentage of enzymatic activity (23). As direct measurement of StAR activity was not routinely available in our clinical practice, classification of the phenotype, diagnosis and therapy could be relied on the clinical presentation together with the known functional consequences of the identified *STAR* variants reported in the literature.

Classic LCAH has the highest prevalence in Japan, Korea, and India, but is quite rare in European populations (18, 23). Only several cases of LCAH have been reported in the pediatric population in Europe (**Table 3**).

### 4.2 Clinical and molecular characterization of the patients

To our knowledge, this is the first report of siblings with LCAH in Serbia, carrying the same compound heterozygous *STAR* genotype: c.749G>A (p.Trp250Ter) and c.562C>T (p.Arg188Cys). The same compound variants were reported in a 46, XY male infant in Switzerland. He presented at age 3.5 months with generalized hyperpigmentation, cryptorchidism, micropenis, but no hypospadias (15).

In our two patients, LCAH was diagnosed in mid-infancy and early childhood. In the case of LCAH, even when StAR loses its function, a StAR-independent steroidogenesis continues in some organs, which explains why PAI manifests after a few months of life (4). It is shown that 14% of the cholesterol import is StAR-independent (24).

The girl presented with the most common clinical signs of PAI: failure to thrive and vomiting. Because cortisol levels did not rise significantly during the electrolyte imbalance, ACTH stimulation test was performed. The inadequate cortisol response confirmed PAI of unknown etiology (**Table 1**). During the first year of life, PAI may be caused by CAH due to *CYP21A2* gene mutation, various syndromes, adrenal dysgenesis and hypoplasia, disorders characterized by ACTH resistance, metabolic conditions with mitochondrial or peroxisomal defects, as well as disorders of cholesterol synthesis (25, 26). The ACTH stimulation test helped exclude CAH, the most common adrenal disorder in the first few months of life. In contrast to LCAH, patients with CAH due to 21-hydroxylase deficiency have elevated levels of 17-hydroxyprogesterone, and in patients with a 46, XX karyotype, virilized external genitalia. The second most common adrenal disorder in early childhood is adrenal hypoplasia, which can be distinguished from LCAH by the presence of hypoplastic adrenals on ultrasound (3). The definitive diagnosis was made using clinical exome sequencing.

In the boy, the major clinical indicators suggesting PAI were significant electrolyte imbalance, low cortisol level, and high PRA during a febrile infection (**Table 2**). Because his older sister had genetically confirmed LCAH, basic laboratory analyses were sufficient to support the diagnosis of PAI. Impaired testicular androgen secretion *in utero* resulted in insufficient prenatal androgenization and affected genital development in the boy. In patients with LCAH, reduced prenatal androgen exposure may lead to complete female external genitalia in 46, XY boys or to milder forms of differences in sex development.

Patients with LCAH typically have low steroid hormone levels and female-appearing external genitalia in both XX and XY karyotypes. Genetic testing identified the same *STAR* gene variants as those found in his sister.

The literature review shows that PAI in non-classical LCAH is usually diagnosed after age 2 years (24) and rarely at puberty (27, 28). Still, other authors have described the onset of non-classical LCAH at 10–12 months or later (5, 29). In the milder form of LCAH, male genitalia are typically normal or show minimal anomalies, such as cryptorchidism and hypospadias, in 46, XY individuals (27). Unlike non-classical LCAH, patients with the classic form demonstrate complete under virilization/feminization of the external genitalia in 46, XY subjects at birth. In contrast, the external genitalia in 46, XX subjects are normal female (5, 15, 27). During adulthood, male patients develop a decline in gonadal function due to hypergonadotropic hypogonadism and abnormal spermatogenesis (28). In contrast, female patients tend to experience spontaneous puberty, but with anovulatory cycles (22).

#### 4.2.1 Clinical phenotypes reported in patients carrying the c.562C>T (p.Arg188Cys) variant in the *STAR* gene

A heterozygous variant c.562C>T (p.Arg188Cys), a missense mutation at exon 5 of the *STAR* gene, results in the replacement of the amino acid Arginine with Cysteine at position 188 in the polypeptide chain of the StAR protein. According to ACMG criteria, the detected variant is classified as pathogenic (11, 15).

The p.Arg188Cys variant has been reported in both homozygous and compound heterozygous genotypes associated with a broad clinical spectrum.

The clinical severity in these patients likely reflects the combined effect of p.Arg188Cys and the variant present on the second allele. These reports support a variable phenotype, ranging from early childhood PAI to later-onset adrenal dysfunction.

Baker et al. reported the p.Arg188Cys variant in two male Pakistani siblings diagnosed with LCAH at 2.2 and 2.8 years of age (27). Both had a 46, XY karyotype and normal male genitalia at birth, with compensated glucocorticoid and mineralocorticoid deficiencies. Functional analysis showed that StAR retained 13.6% of wild-type activity, which was responsible for the non-classic, milder form of LCAH.

The p.Arg188Cys variant was also detected in a 46, XX Italian infant with normal female genitalia, who presented with PAI and classic LCAH at 7 months old (19).

Burget et al. reported a brother and sister who were diagnosed with LCAH in early infancy and were homozygous for the p.Arg188Cys variant (20). The authors emphasized the importance of monitoring gonadal function during puberty and early adulthood in patients homozygous for p.Arg188Cys due to risk of infertility (20).

Lu et al. reported 47 variants in the *STAR* gene that cause non-classical LCAH, highlighting p.Arg188Cys as the most common variant, which accounts for 37.2% of cases (23). Patients with this *STAR* gene variant exhibit a wide range of PAI onset, spanning from birth to adulthood. Among 17 reported patients homozygous for the p.Arg188Cys, phenotypes varied significantly: out of 11 patients with a 46,XY karyotype, nine presented with typical male external genitalia, while two had hypospadias (23).

#### 4.2.2 Clinical phenotypes reported in patients carrying the c.749G>A (p.Trp250Ter) variant in the *STAR* gene

The second heterozygous variant in our patients is c.749G>A (p.Trp250Ter), a stop codon mutation in exon 7 of the *STAR* gene. The p.Trp250Ter variant introduces a premature termination codon in the final exon of the *STAR* gene and is expected to result in a truncated protein with severely impaired function. However, the clinical phenotype cannot be attributed to this variant independently of the variant present on the second allele.

The p.Trp250Ter variant has been reported in patients with both 46, XY and 46, XX karyotypes and in different biallelic genotypes. A homozygous p.Trp250Ter genotype was previously identified in a 46, XY patient with classic LCAH and female external genitalia (12). The variant has also been reported in 46, XX patients, including in compound heterozygosity with another pathogenic *STAR* variant, who presented with adrenal insufficiency early in life and had normal female external genitalia (15, 17).

The phenotype of our patients cannot be attributed to either variant individually but likely reflects the combined effect of the p.Arg188Cys allele, which has documented residual activity, and the predicted loss-of-function p.Trp250Ter allele.

## 5 Conclusion

LCAH is a serious condition that is often not suspected due to its rarity. Genetic analysis confirms a diagnosis of LCAH and provides valuable information for identifying affected family members. Treatment should begin as soon as the diagnosis of PAI is confirmed. Glucocorticoid, mineralocorticoid, and sex hormone therapy are individualized according to the clinical presentation and laboratory findings. Because genotype and phenotype do not always correlate well, therapy and follow-up should be tailored to each patient, with long-term monitoring of adrenal and gonadal function.

## Data Availability

The raw data supporting the conclusions of this article will be made available by the authors, without undue reservation.
